# Hyaluronic Acid Correlates With Bone Metastasis and Predicts Poor Prognosis in Small-Cell Lung Cancer Patients

**DOI:** 10.3389/fendo.2021.785192

**Published:** 2022-01-26

**Authors:** Cong Zhao, Zhiyun Zhang, Xingsheng Hu, Lina Zhang, Yanxia Liu, Ying Wang, Yi Guo, Tongmei Zhang, Weiying Li, Baolan Li

**Affiliations:** ^1^ Department of Cellular and Molecular Biology, Tuberculosis and Thoracic Tumor Research Institute, Beijing Chest Hospital, Beijing, China; ^2^ General Department, Beijing Chest Hospital, Capital Medical University, Beijing, China; ^3^ Department of Medical Oncology, National Cancer Center, National Clinical Research Center for Cancer, Cancer Hospital, Chinese Academy of Medical Sciences and Peking Union Medical College, Beijing, China

**Keywords:** hyaluronic acid, SCLC, bone metastasis, predictive factor, survival rate

## Abstract

**Background:**

Hyaluronan (HA) is one of the essential elements of the extracellular matrix (ECM), involved in the onset of metastasis in various tumors. The interaction and binding of the ligand–receptor HA/cluster of differentiation-44 (CD44) regulate the physical and biochemical properties of the ECM, which correlates with an increased propensity toward metastasis and poor survival outcome. Our study aimed to explore HA for predicting metastasis and survival rate in patients with small-cell lung cancer (SCLC).

**Materials and Methods:**

This prospective cohort study recruited 72 patients with SCLC. Plasma HA and CD44 levels were assayed by enzyme-linked immunosorbent assay (ELISA) for 72 cases before initial systematic treatment (baseline samples), and plasma HA was detected *via* after-2-cycle-chemotherapy (A-2-C-CT) in 48 samples. Logistic regression analysis and the Cox proportional risk model were used to determine the independent predictors of distant metastasis and survival rate of patients.

**Results:**

Baseline plasma HA was notably associated with bone metastasis (BM) [OR (95% CI = 1.015 (1.006–1.024), *p* = 0.001]. Multivariate logistic regression analysis showed that baseline plasma HA was chosen as an independent predictor of BM. Either baseline HA or CD44 or both were associated with BM. Dynamic alteration of HA was notably associated with A-2-C-CT clinical efficacy. Multivariate Cox regression analysis in forward likelihood ratio showed that A-2-C-CT HA was an independent predictor of progression-free survival (PFS) and overall survival (OS).

**Conclusions:**

HA appears to be used as an independent predictive factor for BM, and the dynamic detection of HA can predict prognosis in SCLC patients. The mechanism of the HA/CD44 axis in BM of SCLC deserves further exploration.

## 1 Introduction

Lung cancer is the second most newly diagnosed cancer and the chief cause of cancer death. Small-cell lung cancer (SCLC), characterized by rapid relapse and poor survival, accounts for approximately 10%–15% of lung cancer (1–3). Despite the high response rate to frontline platinum-containing regimens, resistance and relapse after chemotherapy are inevitable for the majority of patients. The median overall survival (OS) of SCLC is only 2–4 months and the 5-year OS is less than 10% (4–7).

In 2014, the National Cancer Institution defined SCLC as “resistant, recalcitrant and highly invasive tumor,” and the scientific framework identifies five initiatives that could make an impact against SCLC including new tools for tissue collection and tumor models that represent distinct phases of the disease and research into factors that define treatment response or resistance (8). In clinical practice, the prognosis of SCLC patients with the same limited stage is very different: some immortality, some quickly progress in concurrent radiochemotherapy, and a few extensive-stage patients live long. There is a lack of effective prognosis markers in SCLC.

Metastasis is a common complication and an important factor, leading to a markedly shortened survival rate compared with non-metastatic SCLC (9–11). Bone metastasis (BM) of SCLC usually occurs early and is common in the progression of the disease, which is harder to detect due to the restrictions in diagnostic techniques. Diagnosis is only made when symptoms common to distant metastasis or an enlarged lesion is present. The best treatment choice may be missed for many patients, thus affecting the prognosis of the patient (12). So far, there has been an urgent need for high specificity and sensitivity prognostic factors to guide clinical decision-making and discover new therapeutic targets.

The extracellular matrix (ECM) supports tumor proliferation, invasion, and metastasis through extensive cross talk (13). Osteopontin (OPN) as a prometastatic secreted protein plays important roles in the regulation of tumor angiogenesis and the cross talk between tumor cells and stromal cells in the bone microenvironment (14). The interaction of OPN with cluster of differentiation-44 (CD44) mediating several signaling network participates in cancer skeletal metastasis through regulating cell–matrix interactions (15). Osteopontin is a useful predictor of bone metastasis and survival (16). The same as OPN, hyaluronan (HA) is mainly an abundant ECM component, and the HA/CD44 axis plays a significant role in a number of biological functions, promoting tumor progression and therapeutic resistance, eventually resulting in poor prognosis (17). Basic studies have indicated that the HA/CD44 axis can lead to tumor cell migration that can promote metastasis formation in various tumors (18–20), which can lead to tumor cell migration in lung cancer (21). The HA/CD44 axis has been associated with poor prognosis and metastasis in NSCLC (22). A diversity of research has shown that serum HA is a prognostic factor of OS (9, 10, 23). Other research indicated that targeting the HA/CD44 signaling pathway could be a promising approach for the prevention and therapy of lung cancer (24).

However, a few relevant real-world clinical studies have been conducted to investigate if the HA/CD44 signaling pathway is associated with metastasis and prognosis in SCLC. Moreover, our studies previously found that CD44^+^ CTC was associated with liver metastasis (LM) and survival in SCLC. Therefore, we aimed to explore whether the HA/CD44 axis can be an effective predictor of metastasis and prognosis in SCLC.

## 2 Materials and Methods

### 2.1 Patients

**Figure 1:**
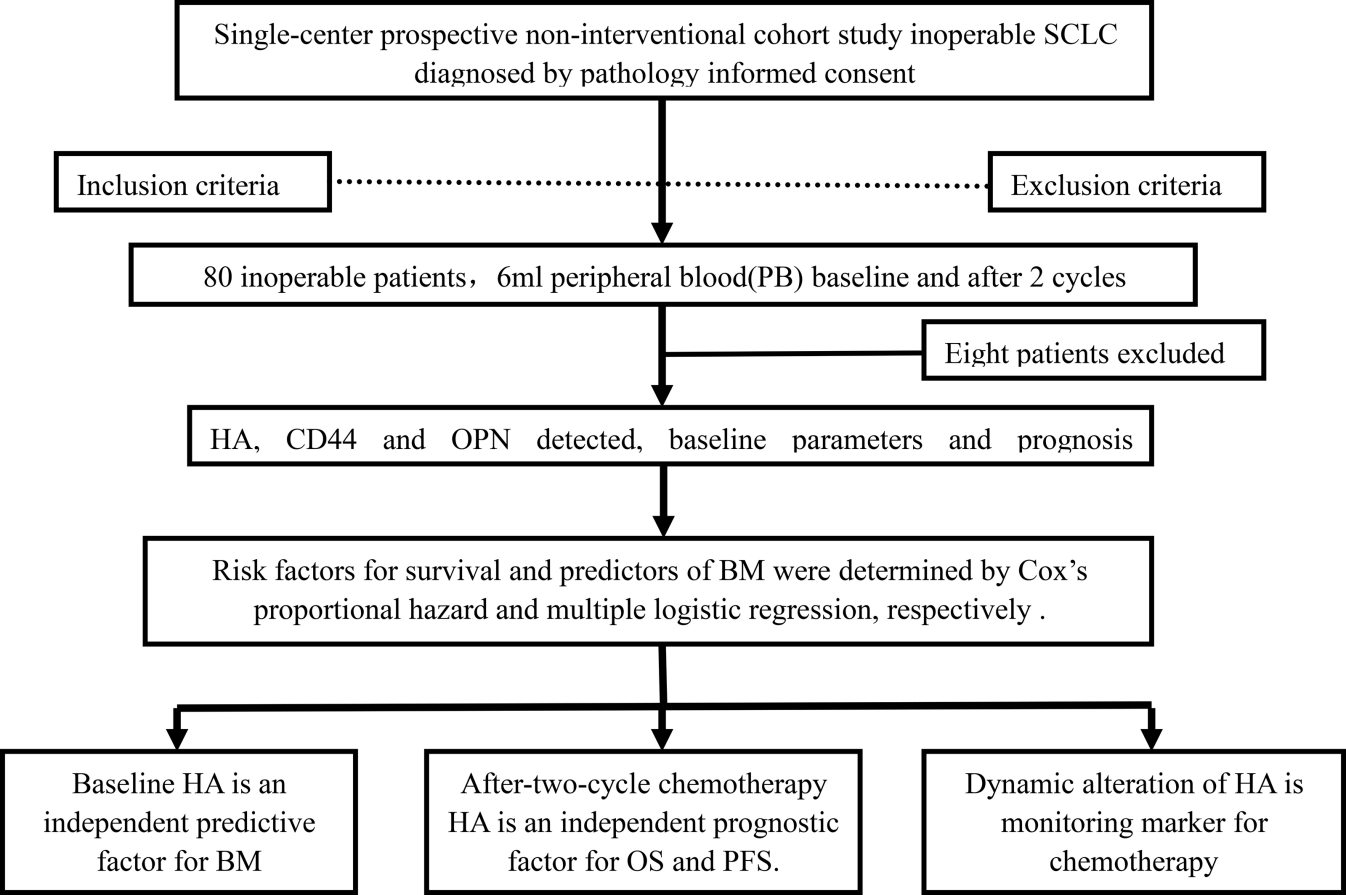
Flowchart of the study design and patient selection.

In this study, 80 consecutive patients diagnosed by pathology or cytology were enrolled, between December 5, 2017, and January 15, 2020. Eight out of 80 cases were excluded due to loss to follow-up, and 72 patients with complete clinical information, who were enrolled in the Beijing Chest Hospital, were included in the study. The flowchart diagram in **Figure 1** illustrates the process of patient enrollment and detection. The included and excluded specimens are listed below.

#### 2.1.1 Inclusion Criteria

Patients were either biopsied by fiber-optic/bronchoscope, endobronchial ultrasound-guided transbronchial needle aspiration (EBUS-TBNA)/mediastinoscopy, or pulmonary puncture under image guidance and then diagnosed with SCLC after a pathological investigation or pathological consultation. The medical records and follow-up cohort data of the patients were completed.

#### 2.1.2 Exclusion Criteria

The age of the patients was <18 years. Patients with severe cardiovascular and/or cerebrovascular diseases or another disease that might have had a significant effect on prognosis were excluded. Patients who had a performance status (PS) >2 were ineligible for this study. Patients who did not complete at least two cycles of chemotherapy for SCLC and those with a history of other malignant tumors were excluded. Patients with psychiatric disorder and patients who had received chemotherapy, radiotherapy, or surgery before transfer to Beijing Chest Hospital, Capital Medical University were also excluded.

### 2.2 Sample Information

A total of 120 frozen plasma samples were included: 72 of them were collected before chemotherapy and 48 of them were drawn after a 2-cycle chemotherapy. All of them were stored in a refrigerator at −80°C.

### 2.3 HA Assay

In total, 120 samples were selected for HA and CD44 ELISA assay, respectively. First, 6 ml of peripheral venous blood was drawn into an ethylenediaminetetraacetic acid (EDTA) anticoagulant tube. Samples were centrifuged for 2 h (3,500 rpm, 5 min) to separate the plasma and were then stored in a refrigerator at −80°C. The levels of plasma HA, CD44, and OPN were detected using human HA-ELISA kits (cat. no. CSB-E04805h, CUSABIO Inc., China), human CD44-ELISA kits (cat. no. ab45912, Cambridge, MA, USA), and human OPN-ELISA kits (cat. no. E-EL-H1347c, Elabscience Biotechnology Inc., China). The evaluation was performed according to the protocol of the manufacturer and based on previous studies (25, 26).

### 2.4 Clinical Stage

All cases were classified according to the combination of TNM classification (8.0) and Veterans Administration Lung Study Group (VALG) staging of the International Union Against Cancer (UICC) and the American Joint Committee on Cancer (AJCC). The treatment plan was performed according to VALG staging.

### 2.5 Evaluation of Therapy Responses

All patients received at least 2 cycles of etoposide combined with platinum (cisplatin or carboplatin), with or without radiotherapy. Tumor response was measured using the Response Evaluation Criteria in Solid Tumors (RECIST version 1.1) (27), including complete response (CR), partial response (PR), stable disease (SD), and progressive disease (PD). Patients evaluated as CR, PR, and SD continued the original chemotherapy regimen, while patients with PD changed the treatment regimen. The objective response rate (ORR) = CR + PR/all patients × 100%.

### 2.6 Follow-Up

A hospital information system (HIS) and telephone were used to collect the data of patients. The basic information of each patient was reviewed in the HIS system after screening according to the inclusion criteria and exclusion criteria. The patients were followed up at 3-month intervals until death or June 30, 2020, whichever came first. Median follow-up was 26.6 (range 5.8–36.3) months. Eight patients were lost to follow-up. The primary endpoint was OS and the secondary endpoints were progression-free survival (PFS), distant metastasis, and therapeutic response. The OS was calculated from the date of SCLC diagnosis to the date of death or the last follow-up. PFS was calculated from diagnosis to the date of progression, death, or the last follow-up.

### 2.7 Statistical Analysis

Data were analyzed using SPSS 22.0, GraphPad Prism 7.0, and R software 4.0. The continuous variables and categorical variables were assessed by Student’s t-test and Pearson’s chi-square, respectively. If the theoretical frequency existing in cells of 2 × 2 tables was less than 5, Fisher’s exact test would be applied. OS and PFS were estimated by the Kaplan–Meier method, with results compared by log-rank tests. Risk factors for survival and predictors of BM were determined by Cox proportional hazard and multiple logistic regression analysis, respectively. The nomogram based on the combined model was established to provide the clinician with a quantitative tool to predict individual probability of BM. To quantify the predictive performance, the area under the curve (AUC), sensitivity, and specificity of the prediction model were obtained from the receiver operating characteristic (ROC) curve analysis; on the other hand, the ROC curve analysis was used to determine optimal cutoff value. A two-tailed *p <*0.05 was considered statistically significant in all analyses.

## 3 Results

### 3.1 Patient Characteristics

We assessed demographic and pretreatment clinical features, including the sex, age, and smoking history of the patients; ECOG-PS; tumor staging according to the eighth edition of the American Cancer TNM Commission System (28); distant metastasis; and the evaluation of clinical efficacy according to RECIST version 1.1 (27). In detail, of the 72 consecutive patients, 56 (77.8%) were males and 16 (22.2%) were females, with a mean age of 60.71 years. The most frequent metastatic site was the bone (25.0%), followed by the liver (18.1%), adrenal glands (8.3%), and brain (4.2%). The major clinicopathological characteristics of the patients are shown in **Table S1**.

### 3.2 Correlation of Baseline Plasma HA With BM

#### 3.2.1 Correlation of Baseline Plasma HA With Distant Metastasis in SCLC

**Figure 2:**
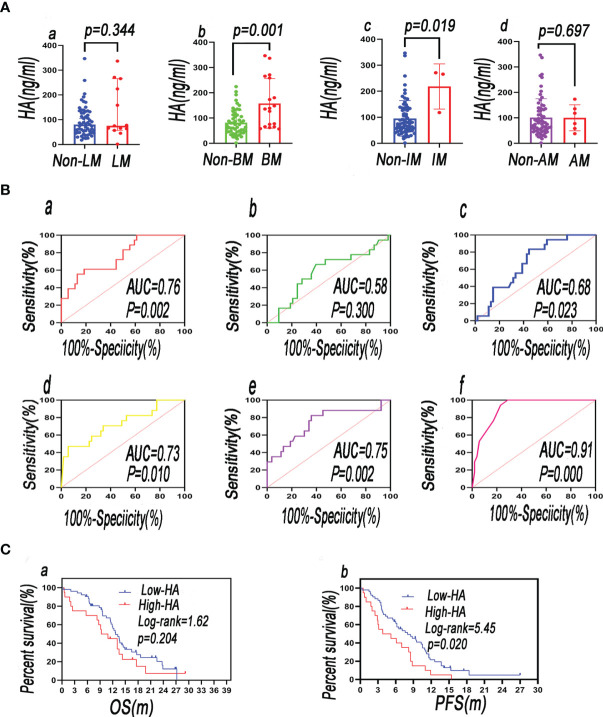
The plasma level of HA in distant metastasis patients vs. that in non-distant metastasis patients—**(Aa)** LM, liver metastasis; **(Ab)** BM, bone metastasis; **(Ac)** IM, intracranial metastasis; **(Ad)** AM, adrenal metastasis. **(B)** ROC curves of blood biomarkers of patients were plotted to analyze their predictive value in BM [**(Ba)** HA; **(Bb)** LDH; **(Bc)** OPN; **(Bd)** pro-GRP; **(Be)** CYFRA21-1; **(Bf)** combination of predictors]. **(C)** Comparing the survival rate between baseline HA^low^ and baseline HA^high^ groups (**a** = OS; **b** = PFS).

The plasma HA level in the intracranial metastasis (IM) group was higher than that in the non-IM group (*p* < 0.05; **Figure 2Ac**). A comparison of the level of HA in the BM group and the non-BM group showed that the level of HA was elevated in the BM group (*p* <0.05; **Figure 2Ab**). According to the other parameters in **Figure 2A**, the difference of HA was not statistically different between the metastasis group and the non-metastasis group (*p* > 0.05) (**Figures 2Ac, d**). In the univariate logistic regression analysis, HA was notably associated with bone [OR (95% CI = 1.015 (1.006–1.024), *p* = 0.001], and the other parameters in **Table S2** did not correlate to HA (*p* > 0.05).

#### 3.2.2 Correlation Between BM and Blood Biomarkers

The relationships between BM and clinicopathological characteristics are shown in **Table S3**. According to the maximum principle of Youden index, the optimal cutoff values were as follows: HA 126.6 ng/ml, CD44 152.5 ng/ml, OPN 135.3 ng/ml, alkaline phosphatase (ALP) 99.0 U/L, lactate dehydrogenase (LDH) 182.0 U/L, carcinoembryonic antigen (CEA) 4.4 ng/ml, neurospecific enolase (NSE) 85.0 ng/ml, pro-gastrin-releasing peptide (pro-GRP) 3,282.0 ng/L, squamous cell carcinoma antigen (SCC) 4.5 μg/ml, and cytokeratin 19 fragment 21-1 (CYFRA21-1) 3.9 μg/ml were calculated by ROC. The high group and low group were divided by their best cutoff values. HA, CD44, OPN, ALP, NSE, pro-GRP, LDH, CEA, and CYFRA21-1 were associated with BM, but SCC did not correlate to BM (*p* = 0.401; **Table S3**). Univariate and multivariate logistic regression analyses were performed on clinical characteristics to determine the predictors of BM. Univariate logistic regression analysis showed that baseline HA, CD44, OPN, ALP, LDH, CEA, NSE, CYFRA21-1, and pro-GRP were related to BM (all *p* < 0.05). According to the results of multivariate logistic regression analysis, baseline HA, LDH, CYFRA21-1, and pro-GRP were chosen as independent predictors of BM, after adjusted for ALP, baseline CD44, CEA, and NSE (**Table 1**).

**Table 1:** Univariate and multivariate logistic regression analyses of variables considered for BM of SCLC patients.

Parameters	Univariate analysis		Multivariate analysis	
	OR (95% CI)	*p*	OR (95% CI)	*p*
Baseline HA				
≥126.0 ng/ml	6.14 (1.93–19.52)	0.020	2.28 (1.06–4.93)	0.036
<126.0 ng/ml	1			
LDH				
>194.5 U/L	3.05 (0.99–9.38)	0.052	6.19 (1.01–37.89)	0.049
≤194.5 U/L	1			
**Pro-GRP**				
>3,282.0 ng/L	14.82 (3.29–66.72)	0.000	16.32 (1.81–147.53)	0.013
≤3,282.0 ng/L	1			
CYFRA21-1				
>3.9 μg/ml	7.11 (1.82–27.79)	0.004	5.702 (1.088–29.880)	0.039
≤3.9 μg/ml	1			

HA, hyaluronic acid; pro-GRP, pro-gastrin-releasing peptide; CYFRA21-1, cytokeratin 19 fragment 21-1; LDH, lactate dehydrogenase; OR, odds ratio; CI, confidence interval.

#### 3.2.3 Predictive Function of Biomarkers for BM Tested by ROC

Based on independent predictors, a predictive model was developed to predict the possibility of BM in newly diagnosed SCLC patients and validated by the ROC. The ROC revealed that HA, LDH, pro-GRP, and CYFRA21-1 were critical for the prediction of BM. The AUCs of HA, LDH, pro-GRP, and CYFRA21-1 were 0.76, 0.58, 0.73, and 0.75, respectively. Their sensitivity and specificity were 69.2% and 84.2%, 66.67% and 60.38%, 53.8% and 94.7%, and 82.35% and 64.15%, respectively. The AUC of their combination was 0.91, and the sensitivity and specificity were 100.0% and 71.15%, respectively (**Figures 2Ba, b, d–f**). The AUC, sensitivity, and specificity of their combination were much better than those of any single indicator.

#### 3.2.4 Comparison of Diagnostic Efficacy of HA and OPN for Bone Metastasis

The AUC of OPN was 0.68, and its sensitivity and specificity were 83.33% and 55.56% (**Figure 2Bc**). The diagnostic efficacy of HA was better than that of the OPN, but there was no statistical difference in ROCs between OPN and HA (DeLong test, *z* = 1.483; *p* = 0.138).

#### 3.2.5 Construction and Validation of a Nomogram

**Figure 3:**
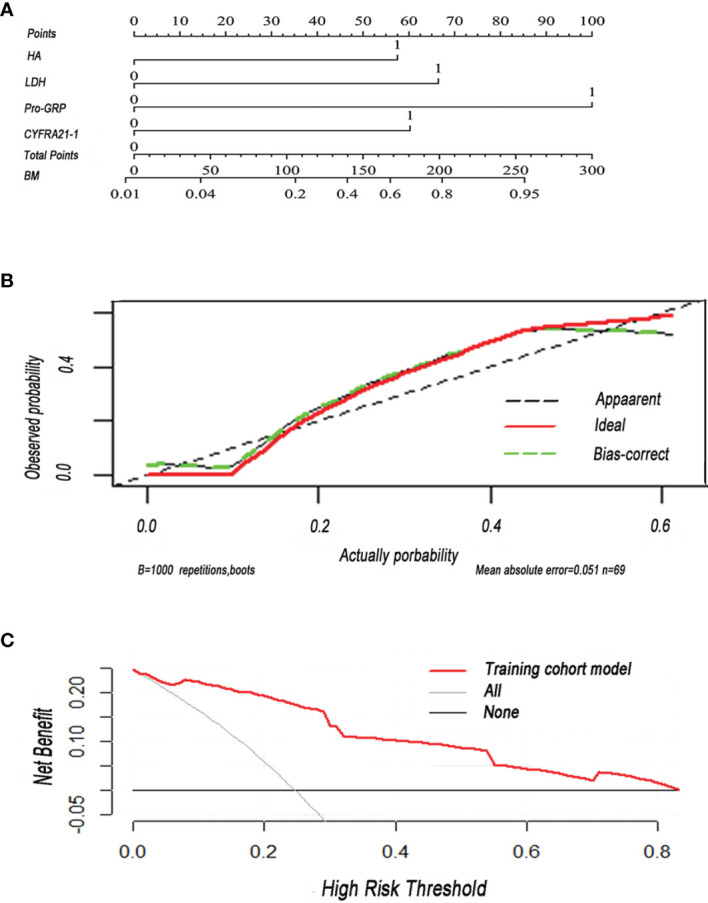
**(A)** Nomogram was plotted for the prediction of BM in SCLC patients. The nomogram incorporated four items (HA, hyaluronan; LDH, lactate dehydrogenase; pro-GRP, pro-gastrin-releasing peptide; CYFRA21-1, cytokeratin 19 fragment 21-1). **(B)** Calibration curve of the nomogram for the prediction of BM. **(C)** Decision curve analysis of the nomogram was conducted to predict the chance of BM in SCLC. Red solid line: prediction model in the training cohort. Gray slash line: assumption that all patients have BM. Solid horizontal line: assumption that no patient has BM.

In order to construct a useful tool to predict BM, we plotted a nomogram on the basis of independent predictors (**Figure 3A**). In the internal validation, the C-index of the nomogram was 0.91 (95% CI: 0.840–0.97). Results showed that all calibration plots closely paralleled the reference prediction line. A departure from the ideal prediction was within an acceptable range when the rate was consistent with BM (**Figure 3B**). The decision curve analysis indicated good positive net benefits in the predictive model and satisfactory potential clinical effects of the predictive model (**Figure 3C**).

#### 3.2.6 Connection of Baseline HA, OPN, and CD44 Correlates With BM

**Figure 4:**
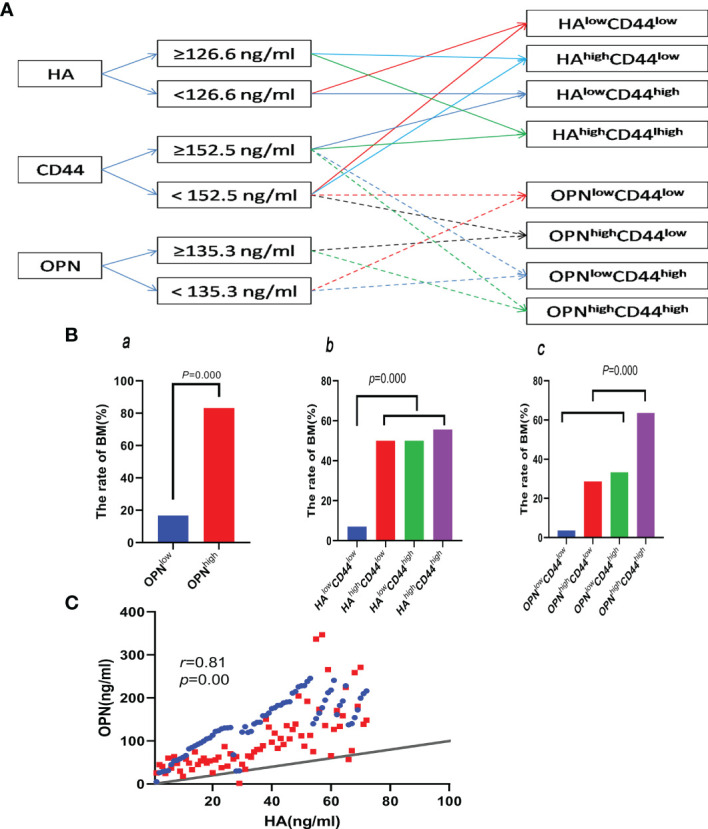
**(A)** Flowchart of the groups by cutoff of HA, OPN, and CD44. **(Ba)** Comparing the rate of BM between the OPN^low^ and OPN^high^ groups. **(Bb, c)** Comparing the rate of BM between the different groups divided by the cutoff of HA, OPN, and CD44 in the plasma. **(C)** Correlation between HA and OPN.

The optimal cutoff values of HA, OPN, and CD44 were 126.6, 135.3, and 152.5 ng/ml, respectively; the high group and low group were divided by their best cutoff values. Patients were grouped in the light of the flowchart (**Figure 4A**). The rate of BM in the OPN^low^ group (16.7%) is lower than that in the OPN^high^ group (83.3%), and the difference was statistically significant (*p* = 0.002) (**Figure 4Ba**). The rate of BM in the HA^low^CD44^low^ group (7.0%) is lower than that in the HA^high^CD44^high^ group (55.6%), HA^high^CD44^low^ group (50.0%), and HA^low^CD44^high^ group (50.0%), respectively (**Figure 4Bb**). The rate of BM in the OPN^high^CD44^high^ group was higher than that in the OPN^low^CD44^high^ group (28.6%) and OPN^high^CD44^low^ group (33.3%), but there was no statistical significance (*p* > 0.05). The rate of BM in the OPN^high^CD44^high^ group (63.6%) was higher than that in the other three groups (18.0%) (*p* < 0.05) (**Figure 4Bc**). HA had a positive correlation with OPN (*r* = 0.81, *p* = 0.00) (**Figure 4C**).

#### 3.2.7 Correlation With Baseline HA and Survival Rate

During the follow-up period, 65 of 72 patients were found with recurrence or metastasis and 55 of 72 patients died of cancer-related causes. Univariate Cox regression analysis was performed and showed that baseline HA was uncorrelated with OS [OR (95% CI) = 1.002 (0.998–1.006), *p* = 0.290] and PFS [OR (95% CI) = 1.003 (0.999–1.006), *p* = 0.111]. Baseline HA was not an independent risk factor of OS and PFS. The median overall survival (mOS) of the baseline HA^low^ group (<126.0 ng/ml) was longer than that of the HA^high^ group (≥126.0 ng/ml) (12.7 vs. 10.10 m, *p* > 0.05) (**Figure 2Ca**). However, there was no statistical significance. The median progression-free survival (mPFS) of the baseline HA^low^ group was longer than that of the baseline HA^high^ group (8.0 vs. 4.5 m, *p* < 0.05), and there was statistical significance. The results showed that poor PFS was associated with baseline elevated HA (**Figure 2Cb**).

### 3.3 Dynamic Change of HA Was Associated With ORR and Survival Rate

#### 3.3.1 Correlation With Dynamic Change of HA and ORR

**Figure 5:**
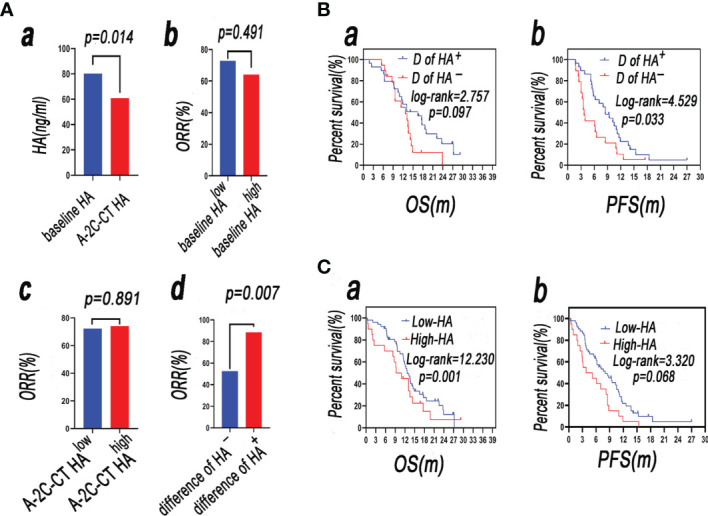
**(Aa)** A-2-C-CT HA was lower than baseline HA (*p* < 0.05); **(Ab)** the ORR in the baseline HA^high^ group was higher than that in the baseline HA^low^ group (*p* < 0.05); **(Ac)** the ORR in the A-2-C-CT HA^low^ group was lower than that in the A-2-C-CT HA^high^ group (*p* > 0.05); **(Ad)** the difference in ORR of the HA^positive^ group was higher than that of the HA^negative^ group (*p* < 0.05). **(B)** Survival analysis of patients with SCLC in the group D of HA^+^ and group D of HA^−^ (**a**: OS, **b**: PFS). **(C)** Survival analysis of patients with SCLC in the A-2-C-CT HA^low^ (named low-HA group) and A-2-C-CT HA^high^ (named high-HA group) groups (**a**: OS, **b**: PFS).

After-2-cycle-chemotherapy (A-2-C-CT) HA was lower than baseline HA [80.21 (48.51–133.32) vs. 60.86 (36.24–79.40), *p* = 0.014] (**Figure 5Aa**). The patients were divided into two groups by the dynamic change of HA, and the method was as follows: firstly, calculating the difference of baseline HA subtracted by A-2-C-CT HA; secondly, determining if the difference of the patient is greater than or equal to 0; enrolling patients according to difference in the HA^positive^ group (difference ≥ 0); and enrolling patients according to difference in the HA^negative^ group (difference < 0). The difference in ORR of the HA^positive^ group was higher than that of the HA^negative^ group (88.5% vs. 52.6%, *p* = 0.007). The dynamic change of HA was correlated with clinical efficacy (**Figure 5Ad**). The ORR in the baseline HA^low^ group was higher than that in the baseline HA^high^ group (72.7% vs. 64.1%, *p* = 0.491), but there was no statistical significance. The ORR in the A-2-C-CT HA^low^ group was lower than that in the A-2-C-CT HA^high^ group (72.2% vs. 74.1%, *p* = 0.891). Baseline HA and A-2-C-CT HA were not correlated with clinical efficacy of the 2-cycle chemotherapy (**Figures 5Ab, c**).

#### 3.3.2 Dynamic Alteration of HA and Survival Rate

Univariate analysis showed that the dynamic alteration of HA was not correlated to OS [OR (95% CI) = 1.758 (0.894–3.459), *p* = 0.102] but correlated to PFS [OR (95% CI) = 1.915 (1.037–3.534), *p* = 0.038]. Multivariate Cox regression analysis in the forward likelihood ratio showed that differences of HA, N stage, TNM stage, NSE, and CEA were chosen as independent predictors of PFS, adjusted by radiotherapy, pro-GRP, CYFRA21-1, IM, BM, LM, M stage, and VALG stage (**Table 2**).

**Table 2:** Univariate and multivariate Cox regression analyses of variables considered for PFS of SCLC patients.

Parameters	Univariate analysis		Multivariate analysis	
	OR (95% CI)	*p*	OR (95% CI)	*p*
D-O-HA			4.467 (1.883–10.595)	0.001
≥0	1.915 (1.037–3.534)	0.038		
<0	1			
N stage				
≤2	2.592 (1.359–4.943)	0.004	2.616 (1.054–6.492)	0.038
>2	1			
TNM stage				
>3	8.173 (3.482–19.184)	0.000	6.519 (2.382–17.843)	0.000
≤3	1			
**NSE**	1.042 (1.017–1.068)	0.001	1.063 (1.019–1.108)	0.004
CEA	1.005 (1.001–1.009)	0.018	1.007 (1.002–1.012)	0.009

D-O-HA, difference of HA; CEA, carcinoembryonic antigen; NSE, plasma neuro-specific enolase; TNM stage, tumor–node–metastasis stage; OR, odds ratio; CI, confidence interval.

#### 3.3.3 Comparison of the Survival Rate of the Difference of the HA^positive^ Group (Difference ≥ 0) and the Difference of the HA^negative^ Group (Difference < 0)

Firstly, the difference of baseline HA subtracted by A-2-C-CT HA was calculated; secondly, the difference of the patient was determined if it was greater than or equal to 0; patients were enrolled according to difference in the HA^positive^ group (difference ≥ 0) (named group D of HA^+^); and the other patients were enrolled according to difference in the HA^negative^ group (difference < 0) (named group D of HA^−^). Survival rate was analyzed by Kaplan–Meier analysis and compared by log-rank test. The mOS of group D of HA^+^ is longer than that of group D of HA^−^ (16.5 vs. 12.7 m, *p* > 0.05) (**Figure 5Ba**). The mPFS of group D of HA^+^ is longer than that of group D of HA^–^ (8.5 vs. 3.7 m), but there was no statistical significance (*p* < 0.05). The results showed that the difference of baseline HA subtracted by A-2-C-CT elevated HA was associated with PFS (**Figure 5Bb**).

### 3.4 Correlation With A-2-C-CT HA and Survival Rate

#### 3.4.1 Univariate and Multivariate Cox Regression Analyses of the Prognostic Factors for OS and PFS

Using OS as the endpoint, ROC analysis was performed to identify the optimal cutoff point of A-2-C-CT HA with the highest sensitivity and specificity. According to the optimal cutoff point (53.1 ng/ml), 48 cases were divided into the A-2-C-CT HA^low^ (≤53.1 ng/ml) and A-2-C-CT HA^high^ (>53.1 ng/ml) groups. **Table S4** shows the relationship between A-2-C-CT HA and various clinical characteristics. The clinicopathological data were compared between the two groups; results indicated that T stage, N stage, M stage, therapeutic segment (TS), pro-GRP, and BM were associated with A-2-C-CT HA (all *p* < 0.05); sex, age, smoking history, illness history, ECOG-PS, LM, clinical efficacy, adrenal metastasis (AM), CEA, NSE, SCC, CYFRA21-1, and IM were not associated with A-2-C-CT HA (all *p* > 0.05) (**Table S4**).

Univariate and multivariate Cox regression analyses were performed on clinical characteristics to determine the predictors of OS. Univariate analysis showed that the factors related to OS were A-2-C-CT HA, sex, N stage, M stage, VALG stage, LM, BM, IM, pro-GRP, and GYFRA21-1 (all *p* < 0.05); multivariate Cox regression analysis in the forward likelihood ratio showed that A-2-C-CT HA [OR (95% CI) = 1.010 (1.002–1.018), *p* = 0.009], sex [OR (95% CI) = 7.361 (1.199–45.171), *p* = 0.031], N stage [OR (95% CI) = 0.257 (0.084–0.782), *p* = 0.017], VALG stage [OR (95% CI) = 27.107 (6.553–112.139), *p* = 0.000], IM [OR (95% CI) = 5.451 (1.204–24.671), *p* = 0.028], pro-GRP [OR (95% CI) = 1.001 (1.000–1.001), *p* = 0.005], and CYFRA21-1 [OR (95% CI) = 1.001 (1.000–1.001), *p* = 0.018] were chosen as independent predictors of OS (**Table 3**). Univariate analysis showed that the factors related to PFS were A-2-C-CT HA, TNM stage, NSE, M stage, N stage, VALG stage, LM, BM, IM, and radiotherapy (*p* < 0.05). Multivariate Cox regression analysis in the forward likelihood ratio showed that A-2-C-CT HA [OR (95% CI) = 1.013 (1.005–1.020), *p* = 0.001], TNM stage [OR (95% CI) = 8.215 (3.207–21.043), *p* = 0.000], and NSE [OR (95% CI) = 1.061 (1.028–1.096), *p* = 0.000] were chosen as independent predictors of PFS (**Table 4**).

**Table 3:** Univariate and multivariate Cox regression analyses of variables considered for OS of SCLC patients.

Parameters	Univariate analysis		Multivariate analysis	
	OR (95% CI)	*p*	OR (95% CI)	*p*
A-2-C-CT HA	2.668 (1.287–5.534)	0.008	1.010 (1.002–1.018)	0.009
Sex	3.388 (1.033–11.105)	0.044	7.361 (1.199–45.171)	0.031
N stage				
≤2	2.475 (1.278–4.794)	0.007	0.257 (0.084–0.782)	0.017
>2	1			
VALG stage				
Extensive	11.222 (4.420–28.493)	0.000	27.107 (6.553–112.139)	0.000
Limited	1			
IM				
Yes	8.053 (2.767–23.435)	0.000	5.451 (1.204–24.671)	0.028
No	1			
Pro-GRP	2.769 (1.349–5.685)	0.006	1.001 (1.000–1.001)	0.005
CYFRA21-1	2.120 (1.009–4.453)	0.047	1.566 (1.079–2.272)	0.018

A-2-C-CT HA, after-2-cycle-chemotherapy hyaluronic acid; pro-GRP, pro-gastrin-releasing peptide; CYFRA2-11, cytokeratin 19 fragment 21-1; VALG, Veterans Administration Lung Study Group; ECOG-PS, Eastern Cooperative Oncology Group performance status; LM, liver metastasis; BM, bone metastasis; IM, intracranial metastasis; OR, odds ratio; CI, confidence interval.

**Table 4:** Univariate and multivariate Cox regression analyses of variables considered for PFS of SCLC patients.

Parameters	Univariate analysis		Multivariate analysis	
	OR (95% CI)	*p*	OR (95% CI)	*p*
A-2-C-CT HA	1.007 (1.002–1.013)	0.008	1.013 (1.005–1.020)	0.001
TNM stage				
>3	8.173 (3.482–19.184)	0.000	8.215 (3.207–21.043)	0.000
≤3	1			
NSE	1.042 (1.017–1.068)	0.001	1.061 (1.028–1.096)	0.000

A-2-C-CT HA, after-2-cycle-chemotherapy hyaluronic acid; NSE, plasma neurospecific enolase; OR, odds ratio; CI, confidence interval.

#### 3.4.2 Comparison of the Survival Rate of A-2-C-CT HA^low^ and A-2-C-CT HA^high^ Groups

Survival rate was analyzed by Kaplan–Meier analysis and compared by log-rank test. The mOS of the A-2-C-CT HA^low^ (named low-HA group) group was longer than that of the A-2-C-CT HA^high^ (named high-HA group) group (18.6 vs. 11.9 m, *p* < 0.05) (**Figure 5Ca**). The mPFS of the low-HA group was longer than that of the high-HA group (10.2 vs. 5.4 m, *p* > 0.05) (**Figure 5Cb**), but there was no statistical significance. The results showed that OS was associated with A-2-C-CT elevated HA.

## 4 Discussion

HA is a ubiquitous component of the ECM, which is known to have essential roles in the growth, migration, and invasion of various cancers (17, 18). HA binding to CD44 plays an important role in regulating cell adhesion, migration, survival, and invasion, through activation of varied signaling pathways like Rho GTPases, Ras-MAPK, and PI3K/AKT pathways (18, 19, 29–33). On the other hand, interfering with the pathway of HA/CD44 can restrict the tumor growth and metastasis (34). El-Mezayen and colleagues suggested that ECM degeneration led to the increase of HA, through HA/CD44-activated downstream pathways, resulting in metastasis (35). We evaluated HA and CD44 in plasma and found that either baseline HA or CD44 or both were associated with BM. Through multivariate logistic regression analysis, we found that HA was an independent predictive factor of BM and shows that HA can predict BM in SCLC stably and reliably.

These results have important clinical significance, because diagnosing BM is still challenging in SCLC. Plasma HA detection is an easy and reproducible means to predict BM in SCLC, which is an important supplement in diagnosing BM in SCLC. In our study, the rate of BM in the HA^high^CD44^high^ group is higher than those of the HA^high^CD44^low^ group, HA^low^CD44^high^ group, and HA^low^CD44^low^ group, respectively. So, we speculated that the HA/CD44 axis was associated with BM in SCLC.

However, HA is not the only ligand of CD44, and the role of CD44 in tumorigenesis is due to its binding to extracellular matrix components, including HA and OPN. Previous studies have reported that OPN secreted from tumor-associated cells increases CD44v6 expression in CR-CSCs by activating the Wnt/beta-catenin pathway, which promotes migration and metastasis (36). OPN was a valuable independent predictor of tumor metastasis and survival in osteosarcoma patients (37). OPN and CD44 overexpression correlated with BM in various cancers (14, 38, 39). OPN might be considered as a potential biomarker to BM diagnosis. Our research found that the diagnostic efficacy of HA was similar to that of OPN. Therefore, it was concluded that HA is an important predictor of BM in SCLC.

In addition, HA, LDH, CYFRA21-1, and pro-GRP were chosen as independent predictors of BM; however, compared with the single factor of pro-GRP, CYRAR21-1, LDH, and HA, we found that combining these biomarkers had higher sensitivity for predicting BM in SCLC. We built a nomogram to identify BM in SCLC to elevate diagnostic efficiency. It provided better discrimination ability than that of nomograms for the other biomarkers of BM (40–42). The prediction model that we have established is of significant help in minimizing patient risk by predicting SCLC with BM.

In the past years, several studies indicated that HA and CD44 could regulate the expression of drug transporters (43) and promote chemoresistance, leading to poor clinical efficacy in a wide spectrum of tumor cell types (44, 45). Wang showed that HA mediated the formation of a complex including CD44 and the epidermal growth factor receptor (EGFR) played major roles in chemoresistance in head and neck squamous cell carcinoma (HNSCC) (45, 46). HA production in ovarian cancer cells was increased in cancer tissues collected following chemotherapy treatment and at recurrence. Furthermore, HA treatment significantly increased the expression of the ATP-binding cassette (ABC) drug transporters (ABCB3, ABCC1, ABCC2, and ABCC3), but only in ovarian cancer cells expressing CD44 (47). In the present study, dynamic alteration of biomarkers after the 2-cycle chemotherapy plays a potential role in antitumor therapy lung cancer (48, 49). Similar to the results of our research, the dynamic change of HA was correlated with clinical efficacy, predicting poor therapeutic response. Detecting the alteration of HA has important clinical significance in SCLC. Because SCLC is sensitive to chemotherapy in the early stage, however, after a 4-cycle-chemotherapy, most cases display chemotherapy resistance and eventually lead to disease progression or recurrence (50). Moreover, as soon as progression or recurrence occurs, we should change therapeutic regimens and re-evaluate the prognosis. In the clinic, alteration of HA may be a powerful tool to test the curative effect and direct doctors to adjust the treatment plan.

Despite decades of basic and clinical research, little progress has been made in finding the predictors of poor outcome in SCLC. HA and its major cell surface receptor, CD44, have been suggested to be important cellular mediators influencing prognosis in several malignant tumors. HA/CD44 interactions with bone morphogenetic protein (BMP) promote BMP4/7-dependent Id1/3 protein expression in melanoma, contributing to reduced survival in melanoma patients (51). Other previous studies reported that HA was a meaningful biomarker for prognosis of various solid tumors (50, 52–54). In our study, results showed the same trend. A-2-C-CT HA was an independent predictive factor of PFS and OS. However, Corte and colleagues reported that a significant association between HA intratumoral levels and relapse-free survival and overall survival in the overall group of patients was not found (55). There are some potential discrepancies between the results of this study and those of the mentioned study, including sample size, histological types, detection method, and testing conditions. The specific mechanism remains unclear and needs to be explored by cell- and animal-based experiments. In summary, A-2-C-CT HA has great significance in the clinic, to predict the survival rate of SCLC.

### 4.1 Limitation

Some limitations of this study should be addressed. First, the sample size of this study was small and may be prone to bias. A multicenter study with a larger sample size is needed to verify the results. Secondly, with a long inclusion period (3 years), a time-trend bias was unavoidable. We tried to limit the possibility of missing information by combining physical and electronic medical charts to obtain quality data and a vigorous analysis of outcomes.

## 5 Conclusions

Our study also provides that baseline HA can be used as an independent predictive factor for BM. Furthermore, the correlation between dynamic alteration of HA and chemotherapy response suggests the clinical value of plasma HA levels as a simple, accurate, and economic treatment monitoring marker which may improve the management of SCLC patients. The level of A-2-C-CT HA may be a novel, minimally invasive, and cost-effective prognostic marker in SCLC. However, the specific molecular mechanisms in SCLC are unclear, and we will try to elucidate the more specific molecular mechanism in this research field.

## Data Availability Statement

The raw data supporting the conclusions of this article will be made available by the authors, without undue reservation.

## Ethics Statement

The studies involving human participants were reviewed and approved by Capital Medical University Beijing Chest Hospital, ethics committee. The patients/participants provided their written informed consent to participate in this study.

## Author Contributions

Conception and design: TZ, WL, and BL. Administrative support: BL. Provision of study materials or patients: CZ, ZZ, LZ, YL, and YG. Collection and assembly of data: XH, LZ, and YW. Data analysis and interpretation: CZ, TZ, WL, and BL. Manuscript writing: all authors. Final approval of the manuscript: all authors.

## Funding

This study was supported by grants from Beijing Municipal Science and Technology Commission (no. Z171100001017038), Tong zhou Liang gao Talents Project (no. YH201920), the Tongzhou District Science and Technology Committee Project (no. KJ2020CX010 to TZ), and the Beijing Municipal Administration of Hospitals Incubating Program (no. PX2017050 to LZ).

## Conflict of Interest

The authors declare that the research was conducted in the absence of any commercial or financial relationships that could be construed as a potential conflict of interest.

## Publisher’s Note

All claims expressed in this article are solely those of the authors and do not necessarily represent those of their affiliated organizations, or those of the publisher, the editors and the reviewers. Any product that may be evaluated in this article, or claim that may be made by its manufacturer, is not guaranteed or endorsed by the publisher.

## References

[1] Zhao Y Zhang X Jin C Yu X Zhang M Cao Y. Efficacy and Safety of Endostatin in Combination With Chemotherapy in Small Cell Lung Cancer: A Phase 2 Single-Arm Multicenter Open-Label Trial. Ann Palliat Med (2021) 10(3):3277–85. doi: 10.21037/apm-21-443 33849112

[2] Wang S Yang L Ci B Maclean M Gerber DE Xiao G. Development and Validation of a Nomogram Prognostic Model for SCLC Patients. J Thorac Oncol Off Publ Int Assoc Study Lung Cancer (2018) 13(9):1338–48. doi: 10.1016/j.jtho.2018.05.037 PMC7678404 29902534

[3] Yang X Wang D Yang Z Qing Y Zhang Z Wang G. CEA is an Independent Prognostic Indicator That Is Associated With Reduced Survival and Liver Metastases in SCLC. Cell Biochem Biophys (2011) 59(2):113–9. doi: 10.1007/s12013-010-9121-0 20945115

[4] Wang Y Zou S Zhao Z Liu P Ke C Xu S. New Insights Into Small-Cell Lung Cancer Development and Therapy. Cell Biol Int (2020) 44(8):1564–76. doi: 10.1002/cbin.11359 PMC7496722 32281704

[5] Lattuca-Truc M Timsit JF Levra MG Ruckly S Villa J Dumas I. Trends in Response Rate and Survival in Small-Cell Lung Cancer Patients Between 1997 and 2017. Lung Cancer (2019) 131:122–7. doi: 10.1016/j.lungcan.2019.03.028 31027688

[6] Siegel RL Miller KD Fuchs HE Jemal A. Cancer Statistics, 2021. CA Cancer J Clin (2021) 71(1):7–33. doi: 10.3322/caac.21654 33433946

[7] Xu C Yuan Q Wang W Chi C Zhang Q Li L. Prognostic Significance of Serum Osteopontin Levels in Small Cell Lung Cancer. BMC Pulm Med (2020) 20(1):235. doi: 10.1186/s12890-020-01242-3 32873264 PMC7466423

[8] A Plan of Attack for Deadly Cancers. Cancer Discovery (2014) 4(9):980. doi: 10.1158/2159-8290.CD-NB2014-114 25185171

[9] Vetrano M Ranieri D Nanni M Pavan A Malisan F Vulpiani MC. Hyaluronic Acid (HA), Platelet-Rich Plasm and Extracorporeal Shock Wave Therapy (ESWT) Promote Human Chondrocyte Regeneration *In Vitro* and ESWT-Mediated Increase of CD44 Expression Enhances Their Susceptibility to HA Treatment. PloS One (2019) 14(6):e0218740. doi: 10.1371/journal.pone.0218740 31251756 PMC6599220

[10] Wang YZ Wu WP Jin J Huang J Li FY Zhang J. [Correlation Analysis of the Prognostic Value of Serum Hyaluronic Acid for Breast Cancer Patients]. Zhonghua Yu Fang Yi Xue Za Zhi (2020) 54(9):993–7. doi: 10.3760/cma.j.cn112150-20200629-00941 32907291

[11] Wu W Chen L Wang Y Jin J Xie X Zhang J. Hyaluronic Acid Predicts Poor Prognosis in Breast Cancer Patients: A Protocol for Systematic Review and Meta Analysis. Med (Baltimore) (2020) 99(22):e20438. doi: 10.1097/MD.0000000000020438 PMC12245377 32481447

[12] Liu F Ke J Song Y. Application of Biomarkers for the Prediction and Diagnosis of Bone Metastasis in Breast Cancer. J Breast Cancer (2020) 23(6):588–98. doi: 10.4048/jbc.2020.23.e65 PMC7779727 33408885

[13] Zuo S Wei M Wang S Dong J Wei J. Pan-Cancer Analysis of Immune Cell Infiltration Identifies a Prognostic Immune-Cell Characteristic Score (ICCS) in Lung Adenocarcinoma. Front Immunol (2020) 11:1218. doi: 10.3389/fimmu.2020.01218 32714316 PMC7344231

[14] Pang X Gong K Zhang X Wu S Cui Y Qian BZ. Osteopontin as a Multifaceted Driver of Bone Metastasis and Drug Resistance. Pharmacol Res (2019) 144:235–44. doi: 10.1016/j.phrs.2019.04.030 31028902

[15] Zhang X Xu W. Neutrophils Diminish T-Cell Immunity to Foster Gastric Cancer Progression: The Role of GM-CSF/PD-L1/PD-1 Signalling Pathway. Gut (2017) 66(11):1878–80. doi: 10.1136/gutjnl-2017-313923 PMC5739856 28348197

[16] Hou X Wu X Huang P Zhan J Zhou T Ma Y. Osteopontin Is a Useful Predictor of Bone Metastasis and Survival in Patients With Locally Advanced Nasopharyngeal Carcinoma. Int J Cancer (2015) 137(7):1672–8. doi: 10.1002/ijc.29540 25824984

[17] Hessmann E Buchholz SM Demir IE Singh SK Gress TM Ellenrieder V. Microenvironmental Determinants of Pancreatic Cancer. Physiol Rev (2020) 100(4):1707–51. doi: 10.1152/physrev.00042.2019 32297835

[18] Futamura N Urakawa H Arai E Kozawa E Ishiguro N Nishida Y. Hyaluronan Synthesis Inhibitor Supplements the Inhibitory Effects of Zoledronic Acid on Bone Metastasis of Lung Cancer. Clin Exp Metastasis (2013) 30(5):595–606. doi: 10.1007/s10585-012-9563-4 23288481

[19] Hiraga T Ito S Nakamura H. Cancer Stem-Like Cell Marker CD44 Promotes Bone Metastases by Enhancing Tumorigenicity, Cell Motility, and Hyaluronan Production. Cancer Res (2013) 73(13):4112–22. doi: 10.1158/0008-5472.CAN-12-3801 23633482

[20] Ringer J Morrison B Kingsley K. Evaluation of Hyaluronic Acid to Modulate Oral Squamous Cell Carcinoma Growth *In Vitro* . J Funct Biomater (2020) 11(4):72. doi: 10.3390/jfb11040072 PMC7711867 33019572

[21] Song JM Molla K Anandharaj A Cornax I MG OS Kirtane AR. Triptolide Suppresses the *In Vitro* and *In Vivo* Growth of Lung Cancer Cells by Targeting Hyaluronan-CD44/RHAMM Signaling. Oncotarget (2017) 8(16):26927–40. doi: 10.18632/oncotarget.15879 PMC5432308 28460475

[22] Najjar F Alammar M Al-Massarani G Almalla N Aljapawe A Ikhtiar A. Circulating Endothelial Cells and Microparticles for Prediction of Tumor Progression and Outcomes in Advanced Non-Small Cell Lung Cancer. Cancer biomark (2017) 20(3):333–43. doi: 10.3233/CBM-170130 28800312

[23] Spadea A Rios de la Rosa JM Tirella A Ashford MB Williams KJ Stratford IJ. Evaluating the Efficiency of Hyaluronic Acid for Tumor Targeting *via* CD44. Mol Pharm (2019) 16(6):2481–93. doi: 10.1021/acs.molpharmaceut.9b00083 31013093

[24] Song JM Im J Nho RS Han YH Upadhyaya P Kassie F. Hyaluronan-CD44/RHAMM Interaction-Dependent Cell Proliferation and Survival in Lung Cancer Cells. Mol Carcinog (2019) 58(3):321–33. doi: 10.1002/mc.22930 PMC11005861 30365189

[25] Niu CC Lin SS Yuan LJ Chen LH Yang CY Chung AN. Correlation of Blood Bone Turnover Biomarkers and Wnt Signaling Antagonists With AS, DISH, OPLL, and OYL. BMC Musculoskelet Disord (2017) 18(1):61. doi: 10.1186/s12891-017-1425-4 28153008 PMC5290649

[26] Dudek AZ Wang X Gu L Duong S Stinchcombe TE Kratzke R. Randomized Study of Maintenance Pemetrexed Versus Observation for Treatment of Malignant Pleural Mesothelioma: CALGB 30901. Clin Lung Cancer (2020) 21(6):553–61 e1. doi: 10.1016/j.cllc.2020.06.025 32727707 PMC7606734

[27] Micke P Faldum A Metz T Beeh KM Bittinger F Hengstler JG. Staging Small Cell Lung Cancer: Veterans Administration Lung Study Group Versus International Association for the Study of Lung Cancer–What Limits Limited Disease? Lung Cancer (Amsterdam Netherlands) (2002) 37(3):271–6. doi: 10.1016/s0169-5002(02)00072-7 12234695

[28] Wu LL Liu X Jiang WM Huang W Lin P Long H. Stratification of Patients With Stage IB NSCLC Based on the 8th Edition of the American Joint Committee on Cancer (AJCC) Staging Manual. Front Oncol (2020) 10:571. doi: 10.3389/fonc.2020.00571 32373536 PMC7186345

[29] Zulauf N Bruggmann D Groneberg D Oremek GM. Expressiveness of Bone Markers in Breast Cancer With Bone Metastases. Oncology (2019) 97(4):236–44. doi: 10.1159/000500675 31412345

[30] Simpson MA Wilson CM Furcht LT Spicer AP Oegema TR Jr. McCarthy JB. Manipulation of Hyaluronan Synthase Expression in Prostate Adenocarcinoma Cells Alters Pericellular Matrix Retention and Adhesion to Bone Marrow Endothelial Cells. J Biol Chem (2002) 277(12):10050–7. doi: 10.1074/jbc.M110069200 11790779

[31] Wang X Du Z Liu X Song Y Zhang G Wang Z. Expression of CD44 Standard Form and Variant Isoforms in Human Bone Marrow Stromal Cells. Saudi Pharm J (2017) 25(4):488–91. doi: 10.1016/j.jsps.2017.04.011 PMC5447408 28579880

[32] Litviakov NV Bychkov VA Stakheeva MN Ibragimova MK Tsyganov MM Gaptulbarova KA. Breast Tumour Cell Subpopulations With Expression of the MYC and OCT4 Proteins. J Mol Histol (2020) 51(6):717–28. doi: 10.1007/s10735-020-09917-1 33037978

[33] Chaudhry GE Akim A Zafar MN Abdullah MA Sung YY Muhammad TST. Induction of Apoptosis and Role of Paclitaxel-Loaded Hyaluronic Acid-Crosslinked Nanoparticles in the Regulation of AKT and RhoA. J Adv Pharm Technol Res (2020) 11(3):101–6. doi: 10.4103/japtr.JAPTR_26_20 PMC7574732 33102192

[34] Roy R Mandal S Chakrabarti J Saha P Panda CK. Downregulation of Hyaluronic Acid-CD44 Signaling Pathway in Cervical Cancer Cell by Natural Polyphenols Plumbagin, Pongapin and Karanjin. Mol Cell Biochem (2021) 476(10):3701–9. doi: 10.1007/s11010-021-04195-1 34081254

[35] El-Mezayen HA Toson el SA Darwish H Metwally FM. Development of a Novel Metastatic Breast Cancer Score Based on Hyaluronic Acid Metabolism. Med Oncol (2013) 30(1):404. doi: 10.1007/s12032-012-0404-8 23275142

[36] Todaro M Gaggianesi M Catalano V Benfante A Iovino F Biffoni M. CD44v6 Is a Marker of Constitutive and Reprogrammed Cancer Stem Cells Driving Colon Cancer Metastasis. Cell Stem Cell (2014) 14(3):342–56. doi: 10.1016/j.stem.2014.01.009 24607406

[37] Liang S Li Y Wang B. The Cancer-Related Transcription Factor Runx2 Combined With Osteopontin: A Novel Prognostic Biomarker in Resected Osteosarcoma. Int J Clin Oncol (2021) 26(12):2347–54. doi: 10.1007/s10147-021-02025-4 34546483

[38] Lee MN Song JH Oh SH Tham NT Kim JW Yang JW. The Primary Cilium Directs Osteopontin-Induced Migration of Mesenchymal Stem Cells by Regulating CD44 Signaling and Cdc42 Activation. Stem Cell Res (2020) 45:101799. doi: 10.1016/j.scr.2020.101799 32339903

[39] Chu JE Xia Y Chin-Yee B Goodale D Croker AK Allan AL. Lung-Derived Factors Mediate Breast Cancer Cell Migration Through CD44 Receptor-Ligand Interactions in a Novel *Ex Vivo* System for Analysis of Organ-Specific Soluble Proteins. Neoplasia (2014) 16(2):180–91. doi: 10.1593/neo.132076 PMC3978398 24709425

[40] Fan Z Huang Z Hu C Tong Y Zhao C. Risk Factors and Nomogram for Newly Diagnosis of Bone Metastasis in Bladder Cancer: A SEER-Based Study. Medicine (2020) 99(42):e22675. doi: 10.1097/MD.0000000000022675 33080711 PMC7571943

[41] Hu C Yang J Huang Z Liu C Lin Y Tong Y. Diagnostic and Prognostic Nomograms for Bone Metastasis in Hepatocellular Carcinoma. BMC Cancer (2020) 20(1):494. doi: 10.1186/s12885-020-06995-y 32487048 PMC7268752

[42] Du F Tang N Cui Y Wang W Zhang Y Li Z. A Novel Nomogram Model Based on Cone-Beam CT Radiomics Analysis Technology for Predicting Radiation Pneumonitis in Esophageal Cancer Patients Undergoing Radiotherapy. Front Oncol (2020) 10:596013. doi: 10.3389/fonc.2020.596013 33392091 PMC7774595

[43] Misra S Ghatak S Toole BP. Regulation of MDR1 Expression and Drug Resistance by a Positive Feedback Loop Involving Hyaluronan, Phosphoinositide 3-Kinase, and Erbb2. J Biol Chem (2005) 280(21):20310–5. doi: 10.1074/jbc.M500737200 15784621

[44] Bourguignon LYW. Matrix Hyaluronan-CD44 Interaction Activates MicroRNA and LncRNA Signaling Associated With Chemoresistance, Invasion, and Tumor Progression. Front Oncol (2019) 9:492. doi: 10.3389/fonc.2019.00492 31293964 PMC6598393

[45] Wang SJ Bourguignon LY. Role of Hyaluronan-Mediated CD44 Signaling in Head and Neck Squamous Cell Carcinoma Progression and Chemoresistance. Am J Pathol (2011) 178(3):956–63. doi: 10.1016/j.ajpath.2010.11.077 PMC3069910 21356346

[46] Toole BP Slomiany MG. Hyaluronan: A Constitutive Regulator of Chemoresistance and Malignancy in Cancer Cells. Semin Cancer Biol (2008) 18(4):244–50. doi: 10.1016/j.semcancer.2008.03.009 PMC2517221 18534864

[47] Ricciardelli C Ween MP Lokman NA Tan IA Pyragius CE Oehler MK. Chemotherapy-Induced Hyaluronan Production: A Novel Chemoresistance Mechanism in Ovarian Cancer. BMC Cancer (2013) 13:476. doi: 10.1186/1471-2407-13-476 24124770 PMC3852938

[48] Ponomaryova AA Morozkin ES Rykova EY Zaporozhchenko IA Skvortsova TE Dobrodeev capital AC. Dynamic Changes in Circulating miRNA Levels in Response to Antitumor Therapy of Lung Cancer. Exp Lung Res (2016) 42(2):95–102. doi: 10.3109/01902148.2016.1155245 26986825

[49] Shi Y Liu X Lou J Han X Zhang L Wang Q. Plasma Levels of Heat Shock Protein 90 Alpha Associated With Lung Cancer Development and Treatment Responses. Clin Cancer Res an Off J Am Assoc Cancer Res (2014) 20(23):6016–22. doi: 10.1158/1078-0432.CCR-14-0174 25316816

[50] Paumier A Le Pechoux C. Radiotherapy in Small-Cell Lung Cancer: Where Should It Go? Lung Cancer (Amsterdam Netherlands) (2010) 69(2):133–40. doi: 10.1016/j.lungcan.2010.04.019 20605651

[51] Wu RL Sedlmeier G Kyjacova L Schmaus A Philipp J Thiele W. Hyaluronic Acid-CD44 Interactions Promote BMP4/7-Dependent Id1/3 Expression in Melanoma Cells. Sci Rep (2018) 8(1):14913. doi: 10.1038/s41598-018-33337-7 30297743 PMC6175841

[52] da Silva MNR Mendes A Martins JRM Tobias-Machado M Pinhal M. Prospective Evaluation of Chondroitin Sulfate, Heparan Sulfate and Hyaluronic Acid in Prostate Cancer. Int Braz J Urol (2018) 44(6):1139–46. doi: 10.1590/S1677-5538.IBJU.2017.0569 PMC6442162 30516927

[53] Peng C Wallwiener M Rudolph A Cuk K Eilber U Celik M. Plasma Hyaluronic Acid Level as a Prognostic and Monitoring Marker of Metastatic Breast Cancer. Int J Cancer (2016) 138(10):2499–509. doi: 10.1002/ijc.29975 26686298

[54] Wu RL Huang L Zhao HC Geng XP. Hyaluronic Acid in Digestive Cancers. J Cancer Res Clin Oncol (2017) 143(1):1–16. doi: 10.1007/s00432-016-2213-5 27535565 PMC11819425

[55] Corte MD Gonzalez LO Lamelas ML Alvarez A Junquera S Allende MT. Expression and Clinical Signification of Cytosolic Hyaluronan Levels in Invasive Breast Cancer. Breast Cancer Res Treat (2006) 97(3):329–37. doi: 10.1007/s10549-005-9130-7 16791488

