# Supplementary material for: Hyaluronic Acid Correlates With Bone Metastasis and Predicts Poor Prognosis in Small-Cell Lung Cancer Patients
Source: Front Endocrinol (Lausanne). 2022 Jan 26;12:785192. doi: 10.3389/fendo.2021.785192 (PMC8826575; doi:10.3389/fendo.2021.785192)
Supplement: Supplementary file 1 [file Table_1.docx]

**Table** S1 Clinicopathological characteristics of SCLC enrolled in the study

| **Parameters** |  | **N(%)/median (IQR)** |
| --- | --- | --- |
| **Sex** |  |  |
|  | Female | 16(22.2%) |
|  | Male | 56(77.8%) |
| **Age** |  | 60.71$\pm10.80$^﹡^ |
| **Smoking history** |  |  |
|  | No | 14(19.4%) |
|  | Yes | 58(80.6%) |
| **ECOG-PS** |  |  |
|  | 0 | 3(4.2%) |
|  | I | 59(81.9%) |
|  | II | 10(13.9%) |
| **VALG stage** |  |  |
|  | Limited | 25(34.72%) |
|  | Extension | 47(62.28%) |
| **TNM stage** |  |  |
|  | ≥4 | 34(47.22%) |
|  | ＜4 | 35(48.61%) |
|  | unknown | 3(4.17%) |
| **Liver metastasis** |  |  |
|  | No | 59(81.9%) |
|  | Yes | 13(18.1%) |
| **Bone metastasis** |  |  |
|  | No | 54(75.0%) |
|  | Yes | 18(25.0%) |
| **Intracranial metastasis** |  |  |
|  | No | 69(95.8%) |
|  | Yes | 3(4.2%) |
| **Adrenal metastasis** |  |  |
|  | No | 66(91.7%) |
|  | Yes | 6(8.3%) |
| **Clinical efficacy** |  |  |
|  | CR+PR | 41(56.94%) |
|  | SD+PD | 20(27.78%) |
|  | unknown | 11(15.28%) |
| **Blood biomarkers** |  |  |
| **HA** (ng/ml) |  | 76.4(49.8-135.1) |
| **OPN**(ng/ml) |  | 140.0(93.0-190.1) |
| **CD44** (ng/ml) |  | 106.10(65.5-152.0) |
| **ALP** (U/L) |  | 85.0(69.0-112.7) |
| **LDH** (U/L) |  | 190.0(151.0-295.0) |
| **CEA** (ng/ml) |  | 4.3(2.3-6.0) |
| **NSE** (ng/ml) |  | 39.6 (26.0-89.3) |
| **Pro-GRP** (ng/L) |  | 1265.5 (471.1-2757.3) |
| **SCC** (ng/ml) |  | 0.2 (0.15 -0.4) |
| **CYFA21-1** (ng/ml) |  | 3.8 (2.8-5.3) |

Abbreviations: ECOG–PS= Eastern Cooperative Oncology Group- Performance Status; TNM stage= Tumor Lymph Node Metastasis stage; VALG stage= Veterans Administration Lung Study Group; HA=hyaluronic acid; ALP =alkaline phosphatase; LDH = lactate dehydrogenase; CEA= carcinoembryonic antigen; NSE= neuro-specific enolase; Pro-GRP = pro-gastrin–releasing peptide; SCC = squamous cell carcinoma antigen; CYFRA21-1=cytokeratin 19 fragments 21-1, IQR = interquartile range.﹡: mean ± standard deviation.
