# Supplementary material for: Hyaluronic Acid Correlates With Bone Metastasis and Predicts Poor Prognosis in Small-Cell Lung Cancer Patients
Source: Front Endocrinol (Lausanne). 2022 Jan 26;12:785192. doi: 10.3389/fendo.2021.785192 (PMC8826575; doi:10.3389/fendo.2021.785192)
Supplement: Supplementary file 2 [file Table_2.docx]

**Table** S2 Association between the level of HA and distant metastasis in SCLC patients

| **Distant metastasis** |  | | **Univariate Logistic Regression Analysis** | |
| --- | --- | --- | --- | --- |
|  | **HA median(IQR)** | ***p*** | **OR(95%CI)** | ***p*** |
| **Liver metastasis** |  | 0.344 |  |  |
| No | 80.1(77.7-132.3) |  | 1.006(.999-1.014) | 0.103 |
| Yes | 75.3(60.1-245.1) |  |  |  |
| **Bone metastasis** |  | 0.001 |  |  |
| No | 66.9(45.1-112.1) |  | 1.015(1.006-1.024) | 0.001 |
| Yes | 260.7(137.4-346.7) |  |  |  |
| **Intracranial metastasis** |  | 0.019 |  |  |
| No | 74.4(48.6-133.1) |  | 1.015(1.002-1.028) | 0.058 |
| Yes | 265.7(118.1-271.3) |  |  |  |
| **Adrenal metastasis** |  | 0.697 |  |  |
| No | 74.8(48.6-134.3) |  | 1.000(0.988-1.012) | 0.986 |
| Yes | 97.8(53.0-145.0) |  |  |  |

Abbreviations: OR= odds ratio; CI= confidence interval, IQR = interquartile range.
