# Supplementary material for: Hyaluronic Acid Correlates With Bone Metastasis and Predicts Poor Prognosis in Small-Cell Lung Cancer Patients
Source: Front Endocrinol (Lausanne). 2022 Jan 26;12:785192. doi: 10.3389/fendo.2021.785192 (PMC8826575; doi:10.3389/fendo.2021.785192)
Supplement: Supplementary file 3 [file Table_3.docx]

**Table** S3 The correlation between BM and blood biomarkers

| **Parameters** | **Groups** | |  | | |
| --- | --- | --- | --- | --- | --- |
|  | **Non-BM group** | **BM group** | **Z/Fisher** | | ***P*** |
| **Baseline HA n (%)** |  |  | 10.56 | 0.001 | |
| ＜126 ng/ml | 43(79.6%) | 7(38.9%) |  |  | |
| ≥126 ng/ml | 11(20.4%) | 11(61.1%) |  |  | |
| **Baseline OPN n (%)** |  |  | 8.224 | 0.004 | |
| ＜135 ng/ml | 30(55.6%) | 3(16.7%) |  |  | |
| ≥135 ng/ml | 24(44.4%) | 15(83.3%) |  |  | |
| **Baseline CD44 n (%)** |  |  | 9.27 | 0.002 | |
| ＜152 ng/ml | 46(83.6%) | 9(50%) |  |  | |
| ≥152 ng/ml | 8(16.4%) | 9(50%) |  |  | |
| **ALP n (%)** |  |  | 6.23 | 0.013 | |
| ≤99u/l | 38(71.7%) | 7(38.9%) |  |  | |
| ＞99u/l | 15(28.3%) | 11(61.1%) |  |  | |
| unknown | 1(1.4%) |  |  |  | |
| **LDH n (%)** |  |  | 3.95 | 0.047 | |
| ≤194.5U/L | 43(60.6%) | 7(35.0%) |  |  | |
| ＞194.5U/L | 28(39.4%) | 13(65.0%) |  |  | |
| unknown | 7(7.1%) |  |  |  | |
| **CEA n (%)** |  |  | 7.00 | 0.001 | |
| ≤4.39ng/ml | 32(60.4%) | 4(23.5%) |  |  | |
| ＞4.39 ng/ml | 21(39.6%) | 13(76.5%) |  |  | |
| unknown | 2(2.8%) |  |  |  | |
| **NSE n (%)** |  |  |  | 0.011 | |
| ≤85.0 ng/ml | 43(81.1%) | 8(47.1%) |  |  | |
| ＞85.0 ng/ml | 10(18.9%) | 9(52.9%) |  |  | |
| unknown | 2(2.8%) |  |  |  | |
| **Pro-GRP n (%)** |  |  |  | 0.000 | |
| ≤3282ng/L | 50(94.3%) | 9(52.9%) |  |  | |
| ＞3282 ng/L | 3(5.7%) | 8(47.1%) |  |  | |
| unknown | 2(2.8%) |  |  |  | |
| **SCC n (%)** |  |  | 0.70 | 0.401 | |
| ≤0.295 ug/mL | 37(69.8%) | 10(58.8%) |  |  | |
| ＞0.295ug/mL | 16(30.2%) | 7(41.2%) |  |  | |
| unknown | 2(2.8%) |  |  |  | |
| **CYFRA21-1 n (%)** |  |  | 9.40 | 0.002 | |
| ≤3.89ug/mL | 32(60.4%) | 3(17.6%) |  |  | |
| ＞3.89 ug/mL | 21(39.6%) | 14(82.4%) |  |  | |
| unknown | 2(2.8%) |  |  |  | |

Abbreviations: HA=hyaluronic acid; ALP =alkaline phosphatase; LDH = lactate dehydrogenase;

CEA= carcinoembryonic antigen; NSE= neuro-specific enolase; Pro-GRP = pro-gastrin–releasing peptide; SCC = squamous cell carcinoma antigen; CYFRA 211= cytokeratin 19 fragment 21-1.
