# Supplementary material for: Hyaluronic Acid Correlates With Bone Metastasis and Predicts Poor Prognosis in Small-Cell Lung Cancer Patients
Source: Front Endocrinol (Lausanne). 2022 Jan 26;12:785192. doi: 10.3389/fendo.2021.785192 (PMC8826575; doi:10.3389/fendo.2021.785192)
Supplement: Supplementary file 4 [file Table_4.docx]

**Table** S4 Clinicopathological characteristics of A-2-C-CT HA^low^ (≤53.10ng/ml) and A-2-C-CT HA^high^(＞53.10 ng/ml) groups

| **Parameters** | | **Groups** | | | | |  |  | | |  | |
| --- | --- | --- | --- | --- | --- | --- | --- | --- | --- | --- | --- | --- |
|  |  | **A-2-C-CT HA^low^**  **(≤53.1ng/ml)** | | **A-2-C-CT HA^high^**  **(＞53.1ng/ml)** | | |  | **Z/Fisher** | | | ***p*** | |
| **Sex** n (%) | |  | |  | | |  |  | | | 1.000 | |
| male | | 17(81.0%) | | 22(81.5%) | | |  |  | | |  | |
| female | | 4(19.0%) | | 5(18.5%) | | |  |  | | |  | |
| **Age** n (%) | |  | |  | | |  | 0.74 | | | 0.390 | |
| ≤62 | | 14(66.7%) | | 21(77.8%) | | |  |  | | |  | |
| ＞62 | | 7(33.3%) | | 6(22.2%) | | |  |  | | |  | |
| **ECOG-PS** n (%) | |  | |  | | |  |  | | | 0.614 | |
| ＜2 | | 20(95.2%) | | 22(88.0%) | | |  |  | | |  | |
| ≥2 | | 1(4.8%) | | 3(12.0%) | | |  |  | | |  | |
| **T stage** n (%) | |  | |  | | |  | 1.91 | | | 0.167 | |
| ≤2 | | 11(52.4%) | | 9(34.6%) | | |  |  | | |  | |
| ＞2 | | 9(45.0%) | | 17(65.4%) | | |  |  | | |  | |
| Unknown | | 2(4.2%) | |  | | |  |  | | |  | |
| **N stage** n (%) | |  | |  | | |  | 1.57 | | | 0.171 | |
| ≤2 | | 14(70.0%) | | 14(51.9%) | | |  |  | | |  | |
| ＞2 | | 6(30.0%) | | 13(48.1%) | | |  |  | | |  | |
| **M stage** n (%) | |  | |  | | |  | 6.86 | | | 0.009 | |
| no | | 15(71.4%) | | 9(33.3%) | | |  |  | | |  | |
| yes | | 6(28.6%) | | 18(66.7%) | | |  |  | | |  | |
| **VALG stage** | |  | |  | | |  | 1.76 | | | 0.151 | |
| limited | | 11(52.4%) | | 9(33.3%) | | |  |  | | |  | |
| extension | | 10(47.6%) | | 18(66.7%) | | |  |  | | |  | |
| **LM** n (%) | |  | |  | | |  |  | | | 0.088 | |
| no | | 19(90.5%) | | 19(70.4%) | | |  |  | | |  | |
| yes | | 2(9.5%) | | 8(29.6%) | | |  |  | | |  | |
| **BM** n (%) | |  | |  | | |  | 6.65 | | | 0.004 | |
| no | | 19(90.5%) | | 14(51.9%) | | |  |  | | |  | |
| yes | | 2(9.5%) | | 13(48.1%) | | |  |  | | |  | |
| **IM** n (%) | |  | |  | | |  |  | | | 0.369 | |
| no | | 20(95.2%) | | 23(85.2%) | | |  |  | | |  | |
| yes | | 1(4.8%) | | 4(14.8%) | | |  |  | | |  | |
| **AM** n (%) | |  | |  | | |  |  | | | 1.000 | |
| no | | 18(90.0%) | | 23(85.2%) | | |  |  | | |  | |
| yes | | 2(10.0%) | | 4(14.8%) | | |  |  | | |  | |
| **Clinical efficacy** |  | |  | | | | | |  |  | | 1.000 |
| CR+PR | 13(72.2%) | | | | 20(74.1%) | | | |  |  | |  |
| SD+PD | 5(27.8%) | | | | 7(25.9%) | | | |  |  | |  |
| unknown | 3(6.25%) | | | |  | | | |  |  | |  |
| **TS** | |  | |  | | |  | 5.00 | | | 0.025 | |
| CE | | 14(70.0%) | | | | 10(37.0%) |  |  | | |  | |
| others | | 6(30.0%) | | | | 17(63.0%) |  |  | | |  | |
| unknown | | 1(2.08%) | | | |  |  |  | | |  | |
| **CEA**(IQR) (ng/ml) | 3.40(2.56-4.79) | | | | 3.51(1.94-10.77) | | | |  | 0.37 | | 0.711 |
| **NSE** (IQR) (ng/ml) | 15.43(12.29-21.21) | | | | 16.77(12.21-21.29) | | | |  | 0.33 | | 0.738 |
| **Pro-GRP** (IQR) (ng/L) | 160.85(51.89-292.03) | | | | 281.45(71.92-1100.00) | | | |  | 1.85 | | 0.064 |
| **SCC**(IQR) (ng/ml) | 0.25(0.21-0.41) | | | | 0.26(0.17-0.34) | | | |  | 1.03 | | 0.304 |
| **CYFRA21-1**(IQR) (ng/ml) | 2.50(2.07-3.71) | | | | 2.99(2.02-3.62) | | | |  | 0.07 | | 0.943 |

Abbreviations: ECOG–PS= Eastern Cooperative Oncology Group- Performance Status; VALG stage= Veterans Administration Lung Study Group; HA=hyaluronic acid; CEA= carcinoembryonic antigen; NSE= neuro-specific enolase; Pro-GRP = pro-gastrin–releasing peptide; SCC = squamous cell carcinoma antigen; CYFRA21-1=cytokeratin 19 fragments 21-1, BM:bone metastasis; LM:liver metastasis; IM: Intracranial metastasis;AM: adrenal metastasis,TS: Theraputic segiment; CE= Carboplatin combined with etoposide OR= odds ratio; CI= confidence interval.
